# Biodegradation of Palm Kernel Cake by Cellulolytic and Hemicellulolytic Bacterial Cultures through Solid State Fermentation

**DOI:** 10.1155/2014/729852

**Published:** 2014-06-12

**Authors:** Mohamed Idris Alshelmani, Teck Chwen Loh, Hooi Ling Foo, Wei Hong Lau, Awis Qurni Sazili

**Affiliations:** ^1^Department of Animal Science, Faculty of Agriculture, Universiti Putra Malaysia (UPM), 43400 Serdang, Malaysia; ^2^Department of Animal Production, Faculty of Agriculture, University of Sebha, Sebha, Libya; ^3^Institute of Tropical Agriculture, Universiti Putra Malaysia (UPM), 43400 Serdang, Malaysia; ^4^Department of Bioprocess Technology, Faculty of Biotechnology and Biomolecular Sciences, Universiti Putra Malaysia (UPM), 43400 Serdang, Malaysia; ^5^Institute of Bioscience, Universiti Putra Malaysia (UPM), 43400 Serdang, Malaysia; ^6^Department of Plant Protection, Faculty of Agriculture, Universiti Putra Malaysia (UPM), 43400 Serdang, Malaysia

## Abstract

Four cellulolytic and hemicellulolytic bacterial cultures were purchased from the Leibniz Institute DSMZ-German Collection of Microorganisms and Cell Culture (DSMZ) and the American Type Culture Collection (ATCC). Two experiments were conducted; the objective of the first experiment was to determine the optimum time period required for solid state fermentation (SSF) of palm kernel cake (PKC), whereas the objective of the second experiment was to investigate the effect of combinations of these cellulolytic and hemicellulolytic bacteria on the nutritive quality of the PKC. In the first experiment, the SSF was lasted for 12 days with inoculum size of 10% (v/w) on different PKC to moisture ratios. In the second experiment, fifteen combinations were created among the four microbes with one untreated PKC as a control. The SSF lasted for 9 days, and the samples were autoclaved, dried, and analyzed for proximate analysis. Results showed that bacterial cultures produced high enzymes activities at the 4th day of SSF, whereas their abilities to produce enzymes tended to be decreased to reach zero at the 8th day of SSF. Findings in the second experiment showed that hemicellulose and cellulose was significantly (*P* < 0.05) decreased, whereas the amount of reducing sugars were significantly (*P* < 0.05) increased in the fermented PKC (FPKC) compared with untreated PKC.

## 1. Introduction

The global consumption of poultry products such as meat or eggs, nowadays, tends to increase in the developing countries. On the other hand, the global demand on the main poultry feedstuffs will be increased, especially the protein and energy resources such as soybean meal and yellow corn, respectively. Therefore, the global price of these feedstuffs will be increased. Thus, the cost of poultry diets will definitely be increased as a result of the global demand. Currently, there are tendencies to use alternative sources of protein and energy to be substituted for soybean meal and yellow corn in monogastric animals such as poultry and swine. It is known that some of the developing countries produce abundant amounts of alternative feedstuffs that are considered agro-waste byproducts such as wheat bran, rice bran, cotton seed meal, copra meal, and palm kernel cake. However, these agro-waste byproducts are featuring on the presence of nonstarch polysaccharides (NSPs) such as xylan and mannan, as well as antinutritional factors in some of these byproducts. It is known that Malaysia is one of the worldwide largest producers of palm oil. The process of oil extraction from palm fruits leads to the production of abundant amounts of palm kernel cake (PKC) which is considered as an agroindustrial byproduct. PKC can be a promising feedstuff for animal feed because of its content of a moderate level of crude protein (14.5–19.24%) and energy [1]. There are two popular methods of oil extraction from palm fruits: by using expeller machine or by using solvent extraction. Therefore, PKC produced by expeller machine contains higher levels of ether extract than those produced by solvent extraction. Based on the method of oil extraction, it contains 4-5%, 0.70–0.90%, and 10–17% of ash, ether extract, and crude fiber, respectively [2]. However, there is limitation of using PKC in monogastric animal diets because of the high levels of crude fiber, coarse texture, and gritty appearance [3–5]. It is also reported that PKC contains 35.2% mannan [6]. It is common to improve the nutritive quality of this byproduct by inoculating cellulolytic and hemicellulolytic microorganisms through SSF. Hence, PKC can be included as feedstuff to substitute yellow corn and soybean meal in the diet of monogastric animals. Thus, the cost of animal production would definitely be decreased as a result of improvement of PKC's nutritive value. It is referred by Fan et al. [6] that PKC has promising amounts of mannose, glucose, and galactose. These reasonable candidates can be used to produce bioethanol through fermentation of microbes [6]. Based on our previous findings [7], the bacterial cultures were characterized in different substrates (carboxymethyl cellulose, xylan from birchwood, and locust bean gum galactomannan). Higher enzyme activities were observed in xylan medium, whereas only mannanase was produced when the bacterial cultures were grown in locust bean gum galactomannan. Thus, different cellulolytic and hemicellulolytic enzymes are needed to degrade the PKC effectively. There is also tendency to characterize bacteria enzymes because of their multienzyme complexes which can provide more function and synergy for biodegrading the agroindustrial waste properly. More focus has been paid in utilizing and improving bacterial enzyme to produce biofuel and bioproduct industries [8]. The technique of SSF can be applied by inoculating one or more of cellulolytic and hemicellulolytic microorganisms to obtain more degradation for the agro-waste byproducts. PKC contains high levels of nonstarch polysaccharides (NSPs) as well as crude fibers. It is mentioned that the concentration of NSPs in PKC was 78% mannan, 3% arabinoxylan, 3% glucuronoxylan, and 12% cellulose [9]. According to the molecular structure of PKC, it was reported that several specific enzymes can be applied to degrade the PKC effectively. These enzymes are mannanase, xylanase, cellulase [10, 11], *α*-galactosidase [5], and *β*-mannosidase [12]. PKC demonstrates high levels of NSPs, and the nutritive quality of this agro-byproduct can be improved by cellulolytic and hemicellulolytic microbes through SSF. Although fungi have many characteristics and produce higher enzyme activity than bacteria, the secondary products from fungi, such as mycotoxins, would depress the growth of animals. The mycotoxin problem can be overcome by replacing fungi with cellulolytic bacteria in SSF [7]. As mentioned above, the most NSPs in PKC are mannan, xylan, and cellulose. Therefore, these bacterial cultures have the ability to produce those enzymes that can break down *β*-glycosidic linkages in NSPs. The other important thing to consider is that these strains are not pathogenic bacteria. Thus, the environment will not be affected during disposing these strains. On the other hand, it was mentioned that* P. polymyxa *exhibited high antimicrobial activity against* Clostridium botulinum*, a major microbiological hazard, in human food. This antimicrobial activity of* P. polymyxa *referred to the production of antimicrobial peptides. The other important thing is that fungi and a wide range of Gram-positive and Gram-negative bacterial cultures, such as* Escherichia coli*,* Streptococcus mutans*,* Leuconostoc mesenteroides*, and* Bacillus subtilis*, were sensitive to the antimicrobial peptides produced by* P. polymyxa *[13, 14]. The combinations of different cellulolytic and hemicellulolytic bacteria in SSF might lead to the production of different types of enzymes at the same time. Hence, the PKC might be degraded effectively, particularly when cellulolytic and hemicellulolytic microbes combined together. Moreover, the synergy of different cellulolytic and hemicellulolytic enzymes can break down different types of *β*-glycosidic linkages in NSPs. Therefore, releasing more sugars improves the nutritive value of PKC. Bacteria belonging to the genus* Bacillus *have been investigated for the production of hemicellulases in different substrates. The characteristics of these bacteria could be attributed to their ability to adhere to the substrate particles and produce filamentous cells in order to penetrate and degrade the substrate effectively. Consequently, the process of SSF was developed by using* Bacillus amyloliquefaciens *DSMZ 1067,* Paenibacillus curdlanolyticus *DSMZ 10248,* P. polymyxa *ATCC 842, and* B. megaterium *ATCC 9885 based on their potential of enzymes activities [7]. All these bacterial cultures are Gram-positive, aerobic, and spore forming rods, except* P. curdlanolyticus *which is considered as facultative anaerobic bacterium. Thus, the objectives of this study were to determine the optimum time for SSF and to improve the nutritive value of the PKC via SSF using single or combined cellulolytic and hemicellulolytic bacteria.

## 2. Materials and Methods

### 2.1. Experiment 1

#### 2.1.1. Organisms and Growth Conditions

Four aerobic cellulolytic and hemicellulolytic bacterial cultures were purchased from DSMZ and ATCC. They were* Bacillus amyloliquefaciens *DSMZ 1067,* Paenibacillus curdlanolyticus *DSMZ 10248,* P. polymyxa *ATCC 842, and* B. megaterium *ATCC 9885. The bacterial cultures were grown in nutrient broth and agar containing (g/L) the following: peptone, 15.0; sodium chloride, 6.0; yeast extract, 3.0; agar-agar, 12.0; and glucose, 1.0 at pH 7.0. The glucose of nutrient broth and agar was substituted with xylan from birchwood as a carbon source. The bacterial cultures were incubated at 30°C and agitated at 200 rpm in a rotary shaker to prepare the working inoculum.

#### 2.1.2. Cellulolytic and Hemicellulolytic Enzymes Activities in SSF within Different Periods of Time

After being ground, sieved, and dried overnight at 60°C, 5 g of PKC was transferred to 150 mL conical flasks. Distilled water was added to the PKC to obtain PKC: moisture ratios of 1 : 0.2, 1 : 0.4, 1 : 0.6, 1 : 0.8, and 1 : 1 (w/v). The process of SSF was followed as described by Alshelmani et al. [7]. After the bacterial cultures were revived (subcultured), the optical density (OD) was adjusted to 1.00 at 600 nm using spectrophotometer. Then, 10% (v/w) inoculum was transferred into each conical flask. The conical flasks inoculated with cellulolytic and hemicellulolytic bacteria were incubated for 12 days under humidified conditions by placing sterile distilled water inside the incubator. One conical flask from each moisture ratio was taken daily in order to extract the crude enzyme, and the enzymes activities were then determined.

#### 2.1.3. Extraction of Crude Enzyme

Extraction of crude enzyme during SSF was accomplished by adding 20 mL sterile distilled water into each conical flask. The flasks were agitated overnight in a rotary shaker at 130 rpm in 25°C. The solution was filtrated using Whatman filter paper number 1 and then centrifuged at 10,000 g, 4°C for 15 min. The supernatant was kept at −20°C for further analysis.

#### 2.1.4. Enzyme Activity Assay

Mannanase, xylanase, and CMCase activities were determined according to the modified methods of Araujo and Ward [15], Bailey et al. [16], and Miller [17], respectively. Briefly, the modified dinitrosalicylic acid (DNS) reagent was employed without the presence of phenol and sodium sulfite. In addition, potassium sodium tartrate was not added separately. The substrates were dissolved in buffers without heating. The incubation time of crude enzyme with substrate was 60 minutes, and 2 mL of DNS reagent was added to stop the reaction. The test tubes were boiled for 5 minutes and cooled under running ice water for another 5 minutes. Standard references were plotted for mannose, xylose, and glucose, and the absorbance was read using a spectrophotometer at 540 nm. The reducing sugars were used as criterion to calculate enzyme activity. The specific enzyme activity was defined as the amount of enzyme that liberates reducing sugar in *μ*mol per min per mg protein under assay conditions.

The soluble protein was determined [18] to calculate the specific enzyme activity for each bacterial culture, and bovine serum albumin (BSA) was used as a standard. All enzyme activities were assayed in triplicate, and the average enzyme activity was presented as *μ*mol/min/mg protein. The enzyme activity was defined as the ability of enzyme to release one *μ*mol of reducing sugar per minute in specific conditions.

### 2.2. Experiment 2

#### 2.2.1. Combinations of Cellulolytic and Hemicellulolytic Bacteria in SSF

After the bacterial cultures were revived (subcultured), the optical density (OD) was adjusted to 1.00 at 600 nm using spectrophotometer. Then, 10% (v/w) inoculum was transferred into each conical flask whether for single or combined bacteria. For instance, 1 mL inoculum was transferred into conical flask containing 10 g PKC for single bacteria, whereas the same inoculums was transferred for the combined bacteria. Fifteen treatment combinations of cellulolytic and hemicellulolytic bacterial cultures were applied for the PKC, and one untreated PKC was considered as a control (16 treatments) in order to improve the nutritive quality of this byproduct (Table 1). Each treatment had four replicates. After the fourth day of SSF, one conical flask was taken from each treatment in order to measure the reducing sugars. SSF lasted for 9 days, and then FPKC samples were autoclaved and dried overnight at 60°C. The samples were kept at −20°C for proximate analysis.

#### 2.2.2. Measurement of Reducing Sugars

An amount of reducing sugars was determined to the FPKC during SSF by cellulolytic and hemicellulolytic bacteria. At the fourth day of fermentation process, one conical flask was taken from each treatment. Then, 20 mL sterile distilled water was added into each conical flask. The flasks were agitated overnight in a rotary shaker at 130 rpm in 25°C. The solution was filtrated using Whatman filter paper number 1 and then centrifuged at 10,000 g, 4°C for 15 min. The supernatant was kept at −20°C for further analysis. Mannose, xylose, and glucose were determined according to the modified methods of Araujo and Ward [15], Bailey et al. [16], and Miller [17], respectively.

#### 2.2.3. Proximate Analysis

Samples of FPKC and untreated PKC (as a control) were analyzed for dry matter (DM), moisture, crude fiber (CF), and crude protein (CP) according to the methods of AOAC [19], whereas acid detergent fiber (ADF), neutral detergent fiber (NDF), and acid detergent lignin (ADL) were determined as described by Goering and Van Soest [20]. Cellulose content was calculated as ADF-ADL, whereas hemicellulose content was calculated as NDF-ADF.

#### 2.2.4. Statistical Analysis

Data were analyzed using one-way analysis of variance (ANOVA), and the treatment means, which showed significant differences at a probability level of 0.05, were separated by Tukey's test using general linear model (GLM) procedure of Statistical Analysis System [21].

## 3. Results and Discussion

### 3.1. Experiment 1

**Figure 1:**
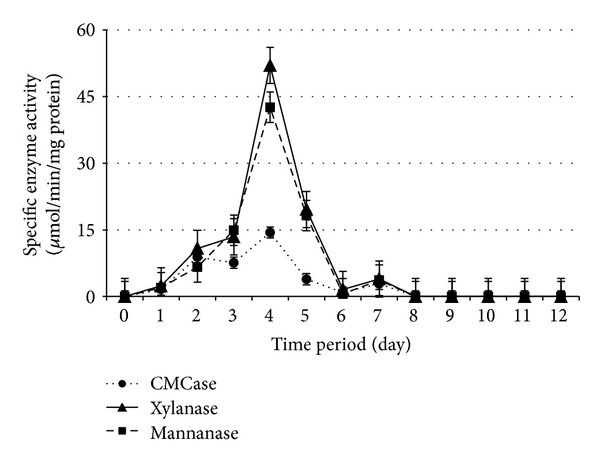
Specific enzyme activity ± SE for* P. polymyxa *under SSF with PKC  : moisture ratio 1 : 0.8 (w/v).

**Figure 2:**
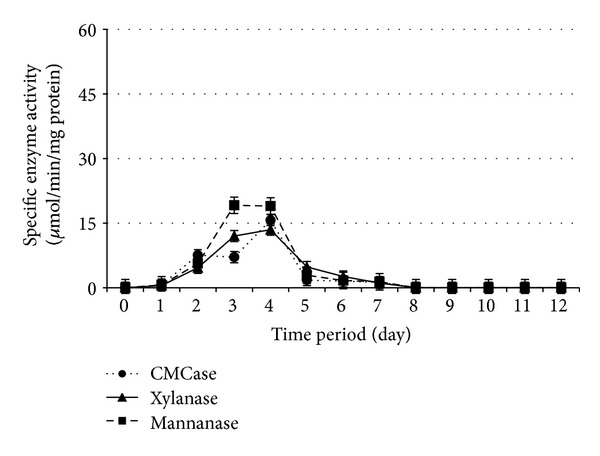
Specific enzyme activity ± SE for* B. megaterium* under SSF with PKC  : moisture ratio 1 : 0.4 (w/v).

**Figure 3:**
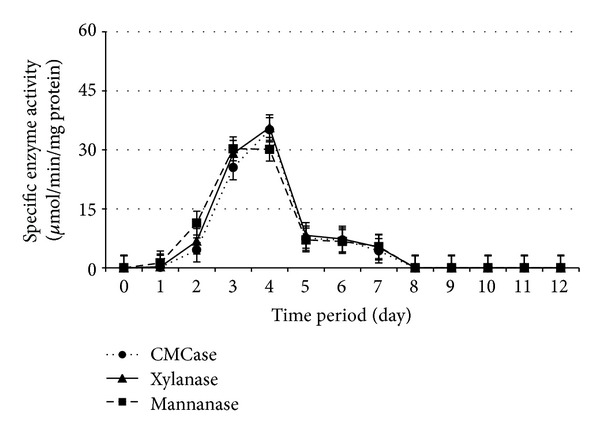
Specific enzyme activity ± SE for* B. amyloliquefaciens* under SSF with PKC  : moisture ratio 1 : 0.6 (w/v).

**Figure 4:**
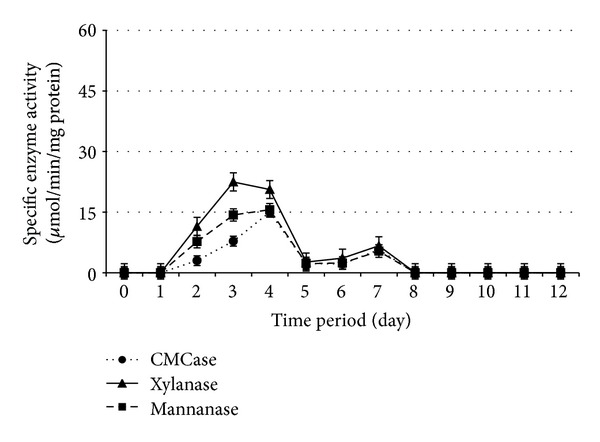
Specific enzyme activity ± SE for* P. curdlanolyticus* under SSF with PKC  : moisture ratio 1 : 1 (w/v).

Cellulolytic and hemicellulolytic enzymes activities during 12 days of SSF by* Paenibacillus polymyxa *ATCC 842,* Bacillus megaterium *ATCC 9885,* B. amyloliquefaciens *DSMZ 1067, and* P. curdlanolyticus *DSMZ 10248 are shown in Figures 1, 2, 3, and 4, respectively. The results obtained showed that the higher enzyme activity was at the fourth day of SSF for the whole bacterial cultures, and the enzyme activity has been decreased to reach zero at the 8th day of SSF. These findings are in agreement with Alshelmani et al. [7], who reported that enzyme activity was dramatically declined for cellulolytic and hemicellulolytic bacteria after 7 days of SSF. The reduction of cellulolytic and hemicellulolytic enzyme activities might be due to the depletion of macro- and micronutrients in the fermentation medium which stressed the bacterial physiology resulting from inactivation of enzyme production [22–24] or may be due to the denaturation of enzymes resulting from variation in pH during SSF [25]. It was observed that* P. polymyxa *ATCC 842 produced higher enzyme activity compared to the other cellulolytic and hemicellulolytic bacteria so that CMCase, xylanase, and mannanase activities were 14.14, 52.00, and 42.60 *μ*mol/min/mg protein, respectively. However, the production of CMCase by* B. amyloliquefaciens *DSMZ 1067 was 35.10 *μ*mol/min/mg protein, higher than the other microbes. These findings are consistent with Mingchao et al. and Anuraj et al. [26, 27] who reported that the ability of* P. polymyxa *to produce multienzyme complexes enabled it to be widely used in agriculture, industry, and environmental processes.

The optimum PKC: moisture ratio appeared to be 1 : 0.8, 1 : 0.4, 1 : 0.6, and 1 : 1 (w/v) during SSF for* Paenibacillus polymyxa *ATCC 842,* Bacillus megaterium *ATCC 9885,* B. amyloliquefaciens *DSMZ 1067, and* P. curdlanolyticus *DSMZ 10248, respectively. These findings are consistent with the results of Gessesse and Mamo [28] who claimed that* Bacillus *sp. produced high enzyme activity when the moisture ratios ranged from 1 : 0.5 to 1 : 1.5 (w/v). The findings are also in agreement with Alshelmani et al. [7] who reported that cellulolytic and hemicellulolytic bacteria being mentioned above exhibited high enzyme activity when the PKC: moisture ratios ranged from 1 : 0.4 to 1 : 1 (w/v). The moisture content in the substrate can be considered as an important factor in SSF as well as microbial growth. A ratio of moisture greater than the optimum level could decrease the porosity of the substrate and lower the oxygen transfer rate. In contrast, a lower moisture level than the optimum ratio could limit the growth of microbes in the substrate [7, 28–32].

### 3.2. Experiment 2

The effects of combinations of different cellulolytic and hemicellulolytic bacterial cultures on nutritive quality of FPKC are shown in Table 2. The SSF process led to the decrease of NDF, ADF, CF, hemicellulose, and cellulose significantly (*P* < 0.05) compared to the untreated PKC, whereas there was no significant effect (*P* > 0.05) on the content of DM, CP, ADL, and ash during SSF. The results obtained showed that the effects of combinations of cellulolytic and hemicellulolytic bacteria were similar to the single microbes in terms of nutritive quality of the FPKC. According to the background of these bacterial cultures, they were capable of producing different types of cellulolytic and hemicellulolytic enzymes. Therefore, it was expected that multiculturing of these microbes under SSF may degrade the PKC more effectively than the single culture. However, the nonstatistically significant differences among these bacteria compared to the single microbes could be attributed to the abilities of these single cultures to produce multienzyme complexes. Another important point to consider is that the optimum PKC: moisture ratios were 1 : 0.8, 1 : 0.4, 1 : 0.6, and 1 : 1 (w/v) during SSF for* P. polymyxa *ATCC 842,* B. megaterium *ATCC 9885,* B. amyloliquefaciens *DSMZ 1067, and* P. curdlanolyticus *DSMZ 10248, respectively. The PKC: moisture ratio employed in current study was 1 : 1 (w/v) during the fermentation process for bacterial combinations. Therefore, the differences among these bacterial cultures in the level of optimum moisture ratios could contribute to the nonsynergistic effect when they combined together. The reduction of NDF, ADF, CF, hemicellulose, and cellulose may be due to the ability of cellulolytic and hemicellulolytic bacteria to produce multi-cellulolytic and hemicellulolytic enzymes [7, 8]. Therefore, these microbes were capable of breaking down *β*-glycosidic linkages in the PKC via their properties of cellulolytic and hemicellulolytic enzymes such as cellulase, xylanase, and mannanase.

These findings are in agreement with Álvarez et al. [10] and Chen et al. [11] who mentioned that cellulolytic and hemicellulolytic enzymes needed to break down the glycosidic bonds in NSPs. The findings are also in agreement with Saenphoom et al. [33] who reported that NDF, ADF, hemicellulose, and cellulose were decreased in PKC treated with cellulolytic enzymes. The release of reducing sugars has been significantly (*P* < 0.05) increased during SSF by cellulolytic and hemicellulolytic bacterial cultures and their combinations compared to the untreated PKC (Table 3).

It is obvious that the amount of reducing sugars released in FPKC by* P. curdlanolyticus *DSMZ 10248 and* P. polymyxa *ATCC 842 was higher compared to the other bacterial cultures and their combinations. For instance, the amount of glucose, xylose, and mannose in FPKC by* P. curdlanolyticus *DSMZ 10248 was 21.75, 20.14, and 27.91 *μ*mol/mL, respectively. These findings are consistent with Alshelmani et al., Pason et al., Waeonukul et al., and Tachaapaikoon et al. [7, 34–37], who indicated that* P. curdlanolyticus *was capable of producing multienzyme complexes. It is also reported that genus* Paenibacillus *was capable of degrading various polysaccharides and was able to produce multifunctional enzymes mainly xylanases [38–40]. The content of soluble protein for* P. polymyxa* ATCC 842,* B. megaterium* ATCC 9885*, B. amyloliquefaciens* DSMZ 1067, and* P. curdlanolyticus *DSMZ 10248 was 0.024, 0.020, 0.009, and 0.025 mg, respectively. The amount of protein in the crude enzyme produced by* B. amyloliquefaciens *was lower than the other bacterial cultures. The amount of protein can affect the specific enzyme activity so that low protein content leads to the increase in specific enzyme activity or* vice versa* [41]. This infers why* P. curdlanolyticus *DSMZ 10248 produced high reducing sugars while its enzyme activities were lower than* B. amyloliquefaciens *DSMZ 1067. In addition, the high enzyme activities produced by* B. amyloliquefaciens *were found when the PKC: moisture ratio was 1 : 0.6 (w/v), whereas the PKC: moisture ratio was 1 : 1 (w/v) when the reducing sugars were determined. The amount of glucose, xylose, and mannose in FPKC by* P. polymyxa *ATCC 842 was 20.83, 18.86, and 27.30 *μ*mol/mL, respectively. These findings are in agreement with Alshelmani et al. [7] who reported that* P. polymyxa *ATCC 842 exhibited higher CMCase, xylanase, and mannanase activities in different substrates. In addition, it was mentioned that* P. polymyxa *was able to produce multienzymes such as *β*-1,3- and *β*-1,6-glucanase [42], cellulase, xylanase, lichenase, and mannanase [43]. No synergistic effect was observed among the combinations of cellulolytic and hemicellulolytic bacterial cultures in terms of nutrient quality of FPKC compared to the single bacterial cultures.

## 4. Conclusions

Based on the current findings, the cellulolytic and hemicellulolytic bacteria mentioned above were capable of degrading the cellulose, hemicellulose, xylans, and mannans molecules. It appears that* P. curdlanolyticus *and* P. polymyxa *were more capable of degrading the PKC effectively. Therefore, the nutritive value of the FPKC has been improved and can be included in the diet of monogastric animals at higher ratios without adverse effects on their growth.

## Figures and Tables

**Table 1:** PKC treated with different combinations of cellulolytic and hemicellulolytic bacteria during SSF∗.

Treatments combinations of bacterial cultures			
Untreated PKC (CON)	A	B	C
D	AB	AC	AD
BC	BD	CD	ABC
ABD	BCD	ABD	ABCD

*A = *P. polymyxa* ATCC 842; B = *B. megaterium* ATCC 9885;

C = *B. amyloliquefaciens* DSMZ 1067; D = *P. curdlanolyticus* DSMZ 10248.

**Table 2:** Effect of combinations of different cellulolytic and hemicellulolytic bacteria on the nutritive quality of PKC during SSF.

Nutrients % (DM basis)									
Bacteria	DM^1^	CP^2^	NDF^3^	ADF^4^	ADL^5^	Hemicel.^6^	Cellulose	CF^7^	Ash
A	92.62	16.77	71.70^c^	47.27^b^	15.43	24.43^b^	31.85^b^	14.09^b^	4.67
B	92.65	16.61	72.64^bc^	47.22^b^	15.25	25.66^b^	31.57^b^	14.12^b^	4.73
C	92.46	16.47	73.18^bc^	47.39^b^	15.54	25.80^b^	31.84^b^	14.33^b^	4.58
D	92.44	16.64	73.54^bc^	47.45^b^	16.04	26.42^b^	31.41^b^	14.29^b^	4.80
AB	92.62	16.60	74.73^b^	48.01^b^	15.25	26.71^b^	32.77^ab^	14.48^b^	4.58
AC	92.81	16.45	74.28^bc^	48.45^ab^	15.43	25.83^b^	33.02^ab^	14.48^b^	5.12
AD	92.72	16.30	73.97^bc^	47.65^b^	14.86	26.31^b^	32.79^ab^	14.71^b^	4.82
BC	92.92	16.62	74.86^b^	47.78^b^	15.20	27.07^b^	32.59^ab^	14.47^b^	4.65
BD	92.73	16.55	73.64^bc^	47.44^b^	14.57	26.20^ab^	32.86^ab^	14.25^b^	4.62
CD	92.73	16.45	73.77^bc^	46.69^b^	14.79	27.09^ab^	31.89^b^	14.36^b^	4.65
BCD	92.88	16.57	73.41^bc^	49.07^ab^	15.49	24.34^b^	33.58^ab^	14.43^b^	4.59
ABC	92.76	16.61	74.57^b^	47.22^b^	16.17	27.01^ab^	31.04^b^	14.70^b^	4.60
ABD	93.00	16.54	73.67^bc^	47.01^b^	15.92	26.32^b^	31.10^b^	14.74^b^	4.52
ACD	92.91	16.73	73.66^bc^	46.34^b^	15.32	26.98^ab^	31.02^b^	14.37^b^	4.49
ABCD	93.03	16.60	72.92^bc^	46.88^b^	15.36	26.03^b^	31.52^b^	14.25^b^	4.57
CON	91.42	16.47	82.29^a^	51.48^a^	15.93	30.81^a^	35.55^a^	16.96^a^	4.74
SEM	0.38	0.12	0.52	0.58	0.41	0.75	0.62	0.19	0.13
Sig.^8^	NS	NS	∗∗	∗∗	NS	∗∗	∗∗	∗∗	NS

^a–c^Means ± SEM. Means with different superscripts in the same column differ significantly (*P* < 0.05).

1: dry matter; 2: crude protein; 3: neutral detergent fiber; 4: acid detergent fiber; 5: acid detergent lignin; 6: hemicellulose; 7: crude fiber; 8: significance; NS: nonsignificant; ∗∗highly significant.

**Table 3:** Effect of combinations of different cellulolytic and hemicellulolytic bacteria on the released reducing sugars in PKC during SSF.

Reducing sugars (*μ*mol/mL)			
Bacteria	Glucose	Xylose	Mannose
A	20.83^b^	18.86^ab^	27.30^ab^
B	18.69^ghi^	17.46^cdef^	25.76^de^
C	18.00^i^	17.14^def^	27.19^abc^
D	21.75^a^	20.14^a^	27.91^a^
AB	19.84^cde^	18.34^bcd^	26.48^bcd^
AC	19.39^defg^	18.03^bcde^	26.49^bcd^
AD	20.65^bc^	19.17^ab^	27.27^ab^
BC	18.29^hi^	16.87^ef^	25.33^e^
BD	18.65g^hi^	17.10^def^	25.92^de^
CD	20.02^bcd^	18.43^bc^	27.06^abc^
BCD	19.63^def^	18.19^bcd^	26.48^bcd^
ABC	19.41^defg^	18.43^bc^	26.11^cde^
ABD	18.98^fgh^	17.48^cdef^	26.53^bcd^
ACD	19.03^efgh^	18.00^bcdef^	26.49^bcd^
ABCD	18.13^i^	16.73^f^	25.25^e^
CON	6.77^j^	7.16^g^	7.67^f^
SEM	0.16	0.24	0.21
Sig.	∗∗	∗∗	∗∗

^a–j^Means ± SEM. Means with different superscripts in the same column differ significantly (*P* < 0.05).

∗∗Highly significant.
